# Melatonin Alleviates the Damage of Polystyrene Microplastics to Porcine Oocytes by Reducing Oxidative Stress and Mitochondrial Damage, and Regulating Autophagy and Apoptosis Levels

**DOI:** 10.3390/ani15213163

**Published:** 2025-10-31

**Authors:** Hui-Mei Huang, Hui-Lin Peng, Chu-Man Huang, Jun-Tong Zhang, Ying-Hua Li, Zi-Li Lin, Qi-Long Cao, Yong-Nan Xu

**Affiliations:** 1 Guangdong Provincial Key Laboratory of Large Animal Models for Biomedicine, South China Institute of Large Animal Models for Biomedicine, School of Pharmacy and Food Engineering, Wuyi University, Jiangmen 529000, China; 2112207086@wyu.edu.cn (H.-M.H.); p0013456@163.com (H.-L.P.); 18318843832@163.com (C.-M.H.); 18026197953@163.com (J.-T.Z.); yhli@wyu.edu.cn (Y.-H.L.); 2 College of Animal Science and Technology, Beijing University of Agriculture, Beijing 102206, China; talzl@163.com; 3 Zhejiang Key Laboratory of Multiomics and Molecular Enzymology, Yangtze Delta Region Institute of Tsinghua University, Jiaxing 314006, China; caoqilong111@126.com

**Keywords:** PS-MPs, melatonin, oocyte maturation, oxidative stress, mitochondrial dysfunction

## Abstract

**Simple Summary:**

Polystyrene microplastics (PS-MPs) are synthetic polymer particles generated during the production and environmental degradation of plastic materials, which tend to accumulate in biological systems and induce toxicological effects. This study investigated the impact of PS-MPs on meiotic maturation in porcine oocytes and the subsequent embryonic developmental competence. The findings demonstrated that PS-MPs exert adverse effects on oocyte quality and embryo development. Supplementation with melatonin during in vitro culture was shown to mitigate these detrimental effects. Therefore, the inclusion of melatonin in culture media may hold potential applications for counteracting reproductive toxicity induced by PS-MPs and could serve as an effective strategy to preserve oocyte quality under conditions of environmental pollutant exposure.

**Abstract:**

Polystyrene microplastics (PS-MPs) are microplastic particles produced during plastic manufacturing and environmental degradation, accumulating over time and entering ecosystems through various pathways, ultimately affecting organisms and inducing toxic effects. Current research on the impact of PS-MPs on mammalian oocyte quality, along with potential preventive mechanisms and strategies to mitigate toxicity, remains limited. This study investigates the effects of antioxidant melatonin on oocyte quality in the presence of PS-MPs, focusing on their influence on oocyte meiotic maturation and embryonic developmental potential. PS-MPs at a concentration of 30 μg/mL significantly impaired first polar body extrusion and reduced the success rate of parthenogenetic activation of mature oocytes in vitro. Furthermore, exposure to PS-MPs exacerbated oxidative stress, mitochondrial dysfunction, apoptosis, and autophagy impairment. Additionally, PS-MPs exposure led to a reduction in antioxidant gene expression and an increase in apoptosis-related gene expression in porcine oocytes. Immunofluorescence assays revealed that PS-MPs may induce oxidative stress, mitochondrial damage, and inflammation through the NF-KB/Nrf2/JNK MAPK signaling pathway crosstalk. Further investigation demonstrated that melatonin supplementation alleviated the toxic effects of PS-MPs exposure, offering potential as a therapeutic approach for mitigating PS-MP-induced reproductive toxicity and preserving oocyte quality.

## 1. Introduction

Polystyrene is a high-molecular material polymerized from styrene monomers. Due to its advantages, such as ease of production and versatility, it is widely used in daily life and industrial applications. However, it is resistant to degradation under natural conditions and can contribute to environmental pollution once discarded [1]. Microplastics (MPs), defined as particles with a diameter of less than 5 mm, include polystyrene MPs (PS-MPs) and other polymers like polyethylene. Recent studies have also indicated that MPs, primarily PS-MPs, can disperse through the atmosphere, contributing to air pollution [2].

A literature search conducted on PubMed and CNKI, using keywords related to MPs and biological toxicity, revealed that MPs and nanoplastics (NPs) pose significant threats to both terrestrial and marine ecosystems, as well as human health. Accumulating evidence highlights the severe toxicity of MP/NPs at various biological levels, including biomolecules, organelles, cells, tissues, organs, and organ systems [3]. Research on MPs in the atmospheric environment has mainly focused on their investigation and adsorption properties, with in vitro biomarker methods used to assess their biological toxicity. Birds are considered effective bioindicators for monitoring atmospheric pollutants, with studies showing biological toxicity in organs such as the lungs, heart, and testes [4,5,6]. In aquatic environments, research on the biological toxicity of PS-MPs primarily concerns aquatic organisms. For example, studies on zebrafish have shown that exposure to PS-MPs increases early developmental toxicity and can even result in toxic effects in offspring through bioaccumulation [7,8,9]. The impact of PS-MPs in soil aligns with findings from atmospheric and aquatic studies.

In recent years, research on mammals, particularly mice, has increased, focusing on the effects of PS-MPs on mammals as a reflection of their potential impact on humans. These studies have revealed that PS-MPs cause biological toxicities in areas such as neurology, respiration, and reproduction [10,11]. The findings suggest that PS-MPs represent a new and escalating environmental pollutant, causing multifaceted harm to biological organisms, with ongoing damage. However, there is still a lack of research on effective prevention and control measures.

Exposure to environmental pollutants and antioxidant compounds can affect oocyte maturation. Nobiletin, a polymethoxyflavonoid compound from citrus fruits, has been shown to significantly increase the percentage of mid-stage mature oocytes, with higher cleavage and blastocyst production rates [10,11]. Thus, antioxidants hold potential for protecting and detoxifying germ cells. Could an antioxidant be identified to mitigate the toxic effects of PS-MPs?

Melatonin (MT), an indoleamine hormone, is a potent antioxidant with potential in mitigating the toxicity of environmental pollutants. The organophosphorus flame retardant 2-ethylhexyldiphenyl phosphate (EHDPP), known for its cytotoxicity, genotoxicity, developmental toxicity, and endocrine-disrupting effects, disrupts mitochondrial distribution and function and DNA integrity and induces apoptosis. Supplementing with MT has been shown to partially alleviate EHDPP-induced oocyte maturation disorders in mice [12]. The high safety profile and efficacy of MT further support its potential for broader human applications.

Mitochondria play a critical role in producing ATP necessary for normal cellular functions. The viability of mature oocytes and subsequent embryos is closely linked to mitochondrial function. Kelli F Malott et al. highlighted the adverse effects of toxic substances on mitochondria and their susceptibility to damage [13]. The balance of reactive oxygen species (ROS) and glutathione (GSH) is crucial for cellular physiological functions and oocyte maturation [14]. Antioxidant compounds can activate the nuclear factor erythroid 2-related factor 2 (Nrf2) pathway, upregulating the expression of superoxide dismutase (SOD) and glutathione peroxidase (GPX) to eliminate excessive ROS, prevent mitochondrial damage, and enhance oocyte quality [15]. Toxic substances can activate the Nrf2 pathway and hinder oocyte maturation by inducing oxidative stress, leading to a decline in oocyte quality in porcine models. MT has been shown to reverse these toxic effects [16].

Autophagy serves not merely to eliminate cellular materials but functions as a dynamic recycling system, generating new components and energy for cellular repair and maintaining homeostasis in vivo [17]. LC3B, a key protein in autophagy, facilitates effective mRNA decay [18]. Apoptosis is a critical process involved in normal cell turnover, embryonic development, and chemically induced cell death [19]. Key apoptotic proteins, such as the pro-apoptotic protein BAX, the anti-apoptotic protein BCL2, and the phospholipid-binding protein Annexin-V, which binds to the cell membrane during early apoptosis, have been identified. Apoptosis plays a central role in tissue homeostasis and affects cell survival [20].

In vitro oocyte injury results in a significant accumulation of ROS within cells, leading to oxidative stress-induced damage to mitochondria and the endoplasmic reticulum. This oxidative damage reduces the developmental potential of oocytes both in vitro and in vivo [21]. When cells are damaged, ROS act as second messengers, activating nuclear factor kappa B (NF-κB) and stress-sensitive kinases, such as c-Jun N-terminal kinases (JNK MAPKs), which increase apoptosis and biological toxicity [22]. Further studies have demonstrated that NF-κB protein levels rise in damaged cells, downregulating the Nrf2/HO-1 pathway, thereby disrupting the antioxidant system and promoting inflammatory and oxidative stress responses [23]. However, the mechanisms underlying ROS-induced injury in porcine oocytes, particularly the crosstalk between the NF-κB, JNK, Nrf2, and HO-1 signaling pathways, remain underexplored.

This study investigated the effects of PS-MPs exposure on the maturation of porcine oocytes and early embryo development, as well as the underlying mechanisms. Understanding the mechanisms of oocyte maturation will inform the development of new intervention strategies aimed at mitigating reproductive damage in mammals caused by environmental pollution, providing a theoretical foundation for studying the biological toxicity of environmental pollutants and raising awareness about environmental protection.

## 2. Materials and Methods

### 2.1. Reagents, Consumables, and Instruments

PS-MPs (HY-148163) were purchased from MedChemExpress (Monmouth Junction, NJ, USA) as a bright yellow aqueous solution with the chemical formula (C_8_H_8_O_3_S)_x_. Unless otherwise specified, all chemicals and reagents were obtained from Sigma-Aldrich (St. Louis, MO, USA).

### 2.2. Vitro Maturation of Oocytes and Expansion of Cumulus Cells

**Figure 1:**
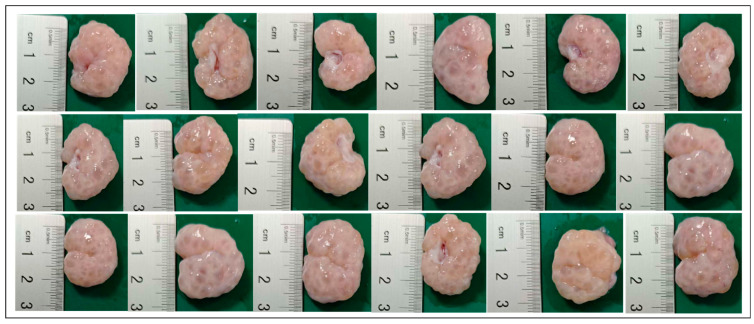
Porcine ovaries containing follicles with diameters of 3 to 6 mm.

The pig ovaries utilized in this study were obtained from sows slaughtered in the morning at a local abattoir. The ovaries were immediately transferred and maintained in a thermos flask containing sterile physiological saline solution at a temperature of 37 °C to preserve tissue viability. Upon arrival at the laboratory, the ovaries were washed three times with sterile normal saline. Follicles with diameters ranging from 3 to 6 mm were collected using a sterile syringe (No. 18 needle) (see Figure 1), which contained oocyte-cumulus complexes (COCs). The COCs were washed five times with a cleaning solution containing 4-(2-hydroxyethyl) piperazine-1-ethanesulfonic acid and N-(2-hydroxyethyl) piperazine-N′-(2-ethanesulfonic acid) (HEPES).

Subsequently, the oocytes were transferred to fresh in vitro maturation (IVM) medium composed of M199 supplemented with 22 μg/mL sodium pyruvate, 10% porcine follicular fluid, 90 μg/mL L-cysteine, penicillin–streptomycin of 1%, 10 IU/mL follicle-stimulating hormone, 20 ng/mL epidermal growth factor, and 10 IU/mL luteinizing hormone (LH). Then, 500 µL of IVM was placed into a four-well culture plate medium in each well, with approximately 55–75 oocytes in each well. To ensure complete immersion of the IVM medium with oocytes, each well was covered with 500 µL of mineral oil. The oocytes were cultured in vitro at 38.5 °C with 5% CO_2_ and 100% humidity for 44–46 h without changing the IVM medium.

### 2.3. Cell Treatment, Parthenogenetic Activation, and In Vitro Embryo Production

To investigate the toxic concentration of PS-MPs, COCs were cultured for 46 h without treatment (control group) or in PS-MPs medium at concentrations of 3, 30, and 300 μg/mL. To identify an effective concentration for alleviation, MT at concentrations of 0, 0.001, 0.01, and 0.1 µM (Sigma-Aldrich, St. Louis, MO, USA) was added to the 30 μg/mL PS-MPs medium, and COCs were treated for 46 h. The degree of cumulus cell dilation and polar body extrusion (PBE) was assessed and recorded under an inverted microscope. Images were analyzed using ImageJ software (NIH, version 1.8.0, National Institutes of Health, Bethesda, MD, USA). A detailed evaluation system of cumulus cell dilation is presented in this study.

COCs were cultured for 46 h without treatment (control group) or in PS-MPs medium at concentrations of 3, 30, and 300 μg/mL. The COCs were blown through a working solution containing 1% hyaluronidase to strip off the outer cumulus layers. Parthenogenetic activation of naked oocytes was performed with two 150 V, 30 μs DC pulse currents in the activation solution, in which the activation solution was made up of 300 mM mannitol, containing 0.5 mM HEPES, 0.05 mM CaCl_2_, 0.1 mM MgSO_4_, and 0.01% polyvinyl alcohol. Activated oocytes were incubated for 3 h in IVC medium containing 7.5 mg/mL cytochalasin B to inhibit the extrusion of dipolar bodies. Finally, the embryos were transferred to IVC medium (bicarbonate-buffered PZM-5 supplemented with 4 mg/mL bovine serum albumin) for 7 days at 37.5 °C, and with 5% CO_2_, in a saturated humidity incubator.

### 2.4. Determination of Reactive Oxygen Species

To measure the level of ROS in porcine oocytes, the ROS assay kit (H2DCFDA, ThermoFisher, Waltham, MA, USA), a cell-permeable probe for detecting intracellular ROS, was employed. After culturing for 44–46 h, 10–20 oocytes, with cumulus cells removed, from each treatment group were incubated at 38.5 °C in an incubator for 30 min with 10 μM H2DCFDA (diluted with PBS-PVA). After washing with PBS-PVA, the porcine oocytes were mounted on slides and imaged under an inverted fluorescence microscope (Ti2, Nikon, Tokyo, Japan). Each sample’s fluorescence intensity was measured with consistent scanning settings, and the intensity of fluorescent pixels was analyzed using ImageJ software (NIH, Bethesda, MD, USA). (Remarks: FITC; excitation wavelength: 465–495 nm, emission wavelength: 512–558 nm.)

### 2.5. Determination of Glutathione

To assess the GSH level in porcine oocytes, the GSH content was measured using 10 µM 4-chloromethyl-6, 8-difluoro-7-hydroxycoumarin (CMF2HC, ThermoFisher Scientific). After 44–46 h of culture, 10–20 oocytes with cumulus cells removed were incubated at 38.5 °C for 30 min with 10 μM H2DCFDA (diluted with PBS-PVA). Following washing with PBS-PVA, the samples were fixed on slides and imaged under an inverted fluorescence microscope (Ti2, Nikon, Tokyo, Japan). The same scanning settings of the fluorescence intensity were measured, and the intensity of the fluorescent pixels was analyzed using ImageJ software (NIH, Bethesda, MD, USA). (Remarks: DAPI; excitation wavelength: 362–396 nm, emission wavelength: 432–482 nm.)

### 2.6. Determination of Mitochondrial Distribution

To examine the mitochondrial content in porcine oocytes, 0.01 mM Mito-Tracker Red (CMXRos, Beyotime, Shanghai, China) was used to evaluate the distribution and quantity of mitochondria. After culturing for 44–46 h, 10–20 oocytes with cumulus cells removed from each group were incubated with 0.01 mM Mito-Tracker Red (diluted with IVM medium) at 38.5 °C in an incubator for 30 min. After washing with PBS, the porcine oocytes were fixed on slides and imaged under an inverted fluorescence microscope (Ti2, Nikon, Tokyo, Japan). The same scanning settings of the fluorescence intensity were measured, and the intensity of fluorescent pixels was analyzed using ImageJ software (NIH, Bethesda, MD, USA). (Remarks: Tx Red; excitation wavelength: 540–580 nm, emission wavelength: 600–660 nm.)

### 2.7. Determination of ATP Content

To assess the ATP level in porcine oocytes, after 46 h of culture, 10–20 oocytes with cumulus cells removed from each treatment group were incubated at 38.5 °C in an incubator for 30 min with 100 μM ATP detection reagent (Beyotime). Following washing with PBS, the porcine oocytes were fixed on slides and imaged under an inverted fluorescence microscope (Ti2, Nikon, Tokyo, Japan). (Remarks: FITC; excitation wavelength: 465–495 nm, emission wavelength: 512–558 nm.)

The same scanning settings of the fluorescence intensity were measured, and the intensity of fluorescent pixels was analyzed using ImageJ software (NIH, Bethesda, MD, USA).

### 2.8. Determination of Annexin-V Content

Annexin-V, a Ca^2+^-dependent phospholipid-binding protein with a molecular weight of 35–36 kDa, binds to the cell membrane of early apoptotic cells where phosphatidylserine is exposed on the outer leaflet. After 46 h of culture, 10–20 oocytes, with cumulus cells removed, from each treatment group were processed according to the Annexin-V kit protocol (BD Biosciences Pharmingen, San Diego, CA, USA; Batch no. 3031146). The oocytes were incubated at 38.5 °C in an incubator for 30 min. Following washing with PBS, imaging was performed using an inverted fluorescence microscope (Ti2, Nikon, Tokyo, Japan). (Remarks: FITC; excitation wavelength: 465–495 nm, emission wavelength: 512–558 nm.)

Consistent scanning settings of fluorescence intensity were measured, and the intensity of fluorescent pixels was analyzed using ImageJ software (NIH, Bethesda, MD, USA).

### 2.9. Detection of Blastocyst Apoptosis by the TUNEL Method

Embryos were incubated for 30 min in 4% paraformaldehyde (Millipore, Burlington, MA, USA) and 1% Triton X-100 (Sigma Aldrich, St. Louis, MO, USA). After washing with PBS three times, the fixed embryos were stained with TUNEL for 1 h using the TUNEL kit (Beyotime, China). After another three washes with PBS, cell nuclei were labeled with 10 mL of VECTASHIELD anti-fluorescence quenching mounting medium containing DAPI reagent. The slides were then sealed with colorless, odorless nail polish.

Images were captured using a fluorescence microscope, and green/red fluorescence intensities were analyzed using ImageJ software. The proportion of TUNEL-positive cells was determined through double staining of TUNEL (green) and DAPI (blue). (Remarks: Tx Red; excitation wavelength: 540–580 nm, emission wavelength: 600–660 nm. FITC; excitation wavelength: 465–495 nm, emission wavelength: 512–558 nm. DAPI; excitation wavelength: 362–396 nm, emission wavelength: 432–482 nm.)

### 2.10. Immunofluorescence

The cumulus–oocyte complex was cultured for 46 h, after which cumulus cells were removed. The samples were washed there times with PBS-PVA, followed by a 30 min incubation in 3.7% paraformaldehyde at room temperature. The samples were permeabilized with 0.3% Triton X-100 at room temperature for 30 min, then placed in PBS-PVA containing 3% BSA and sealed at 37 °C for 1 h. Oocytes were subsequently incubated overnight at −4 °C with the following primary antibodies: rabbit anti-LC3b antibody (#ab48394, Abcam, Cambridge, UK, 1:2000), rabbit anti-Nrf2 antibody (#3177, Cell Signaling Technology, Boston, MA, USA, 1:300), rabbit anti-recombinant JNK1 + JNK2 + JNK3 antibody [EPR16797-211] (#ab179461, Abcam, Cambridge, UK, 1:100), rabbit anti-HO-1 antibody (#86806S, CST, Boston, MA, USA, 1:100), and rabbit anti-NF-κB p65 (D14E12) XP^®^ rabbit mAb antibody (#8242T, Abcam CST, Boston, MA, USA, 1:100). After washing five times with PBS-PVA, the oocytes were incubated with goat anti-rabbit IgG antibody (#ab150077, Abcam, 1:1000) in the dark at 37 °C for 1 h. To label cell nuclei, the oocytes were treated with 10 mL of VECTASHIELD anti-fluorescence quenching mounting medium containing DAPI reagent. (Remarks: Tx Red; excitation wavelength: 540–580 nm, emission wavelength: 600–660 nm. FITC; excitation wavelength: 465–495 nm, emission wavelength: 512–558 nm. DAPI; excitation wavelength: 362–396 nm, emission wavelength: 432–482 nm.)

Following processing, the slides were sealed with colorless, odorless nail polish. Using a fluorescence microscope to capture images, and the green/red fluorescence intensities were analyzed using ImageJ software.

### 2.11. Reverse Transcription–Polymerase Chain Reaction (RT-PCR)

The cells were cultured in a carbon dioxide incubator at 38.5 degrees Celsius with IVM medium for 2 days, and total RNA was isolated using the Dynabeads mRNA Direct Purification Kit (Invitrogen, Carlsbad, CA, USA). RNA was reverse transcribed into cDNA using the SuperScript III First Strand cDNA Synthesis Kit (Invitrogen). Gene expression analysis was performed using TB Green^®^ Premix Ex Taq™ (Tli RNaseH Plus) (Takara Bio, Kusatsu, Japan). The total sample volume was 20 µL, which included 1 µL (10 pmol) of each gene-specific primer, 10 µL of TB Green^®^ Premix Ex Taq™, 7 µL of deionized water, and 1 µL of cDNA sample. The quantitative PCR conditions were as follows: polymerase activation at 95 °C for 180 s, followed by 40 cycles of denaturation at 95 °C for 3 s, annealing at 60 °C for 30 s, extension at 72 °C for 20 s, and a final extension at 72 °C for 5 min. The normalization used GAPDH transcript levels. Gene expression was quantified using the 2^−∆∆Ct^ method. Each cDNA sample was analyzed in triplicate, and three replicate experiments were conducted for each sample. Table 1 lists the primers used in RT-qPCR.

### 2.12. Statistical Analysis

At least three independent experiments were performed for each group. Data are presented as the mean ± SEM. Normalization and analysis were conducted as follows: for each experiment, the control group’s average value was first calculated, and then all control group values were normalized by dividing each by the control group’s average. In a similar manner, the values for each treatment group were normalized by dividing them by the average of the control group. This process yielded standardized values for the treatment groups.

The normalized data were analyzed using SPSS software (v26.0, IBM Corporation, Armonk, NY, USA). Statistical comparisons between groups were performed using the *t*-test or one-way analysis of variance (ANOVA). The *t*-test was used for comparisons between two groups, while one-way ANOVA was used for comparisons among three groups. Results were deemed statistically significant when *p* < 0.05, with * *p* < 0.05 indicating a significant difference, ** *p* < 0.01 indicating a very significant difference, and *** *p* < 0.001 indicating an extremely significant difference. “ns” indicates no significant difference.

## 3. Research Results

### 3.1. Exposure to PS-MPs Affects the Maturation of Porcine Oocytes

**Figure 2:**
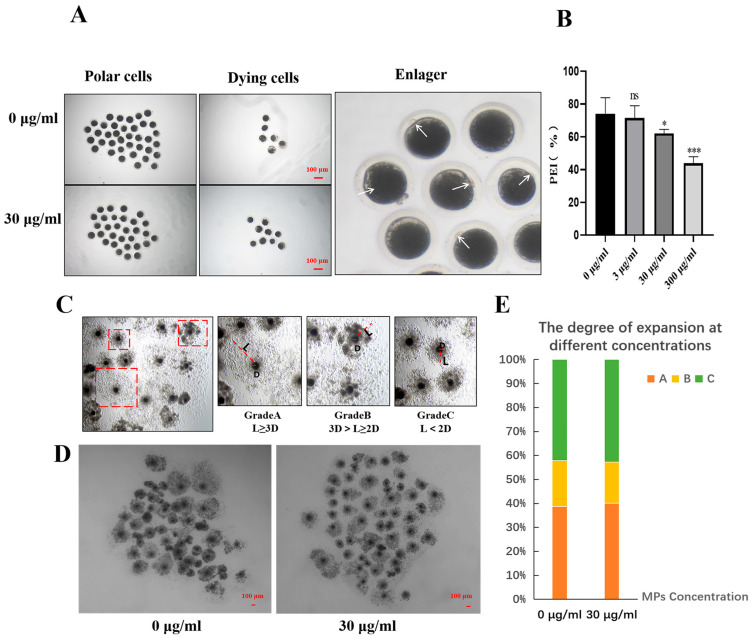
Porcine oocyte maturation was affected when it was exposed to PS-MPs. (**A**) Typical configuration of oocyte maturation after exposure to control and 30 µg/mL PS-MPs. The arrow indicates the PBI. Scale = 100 µm. The enlarged image with white arrow highlights the distinct polar body, used as the standard for polar body selection. The magnification box denotes the area of the image for zooming. (**B**) Polar body extrusion rates in each control group (0, 3, 30, and 300 µg/mL PS-MPs concentrations). “ns” shows no significant difference, * *p* < 0.05, *** *p* < 0.001. (**C**) Typical images of three COCs with varying degrees of expansion. The red dashed box indicates the different levels of expansion, where D represents the oocyte radius and L represents the radius of cumulus expansion. Scale = 100 µm. (**D**) Representative images of cumulus expansion in the control group and the 30 µg/mL PS-MPs group. (**E**) Statistical graphs of cumulus cell dilation in the control group and PS-MPs at different grades. The experiment was repeated three times for each group of data.

To investigate the effect of the environmental pollutant PS-MPs on the meiotic maturation of mammalian oocytes, different concentrations (3, 30, and 300 µg/mL) of PS-MPs were applied, and the morphology of porcine oocytes under a microscope was observed and statistically analyzed by SPSS and ImageJ, with a focus on PBE. As shown in Figure 2A, compared to the control group, the PS-MPs exposure led to varying results. After 46 h of IVM treatment, the meiotic maturation (M II stage) of porcine oocytes significantly decreased with higher PS-MPs concentrations: control group (74.11 ± 9.77%, *n* = 55 oocytes) vs. 3 µg/mL PS-MPs (74.27 ± 5.74%, ns, no significant difference, *n* = 50 oocytes), 30 µg/mL PS-MPs (59.43 ± 2.67%, *p* < 0.05, significant, *n* = 56 oocytes), and 300 µg/mL PS-MPs (43.89 ± 3.94%, *p* < 0.001, extremely significant, *n* = 56 oocytes), *n* = 3. These results indicate that PS-MPs hinder the meiotic maturation of porcine oocytes, suggesting PS-MPs as a potential cytotoxic substance for germ cells. Consequently, 30 µg/mL was selected as the appropriate concentration for subsequent experiments. Cumulus cells, which are closely linked to oocytes, transfer nutrients and signals through specialized spaces. The expansion of cumulus cells was observed and statistically analyzed to further evaluate the effect of PS-MPs on oocyte developmental ability. The expansion of COCs was visually assessed by quantifying the expansion degree of cumulus cells. The diameter of oocytes (denoted as D) and the diffusion diameter of cumulus cells (denoted as L) were measured. The relative dilation (L/D) of cumulus cells in the PS-MPs exposure and control groups was classified into three categories: grade A (complete dilation), grade B (partial dilation), and grade C (no dilation). As shown in Figure 2D, no significant difference in cumulus cell dilation was observed between the groups (0 µg/mL: Grade A dilation: X = 38.71%; B-level expansion X = 19.12%; C-level expansion: X = 42.17%; *n* = 3; 30 µg/mL: Grade A dilation: X = 40.05%; B-level expansion X = 17.21%; C-level expansion: X = 42.74%; *n* = 3).

### 3.2. PS-MPs Exposure Affects Mitochondrial Function, Redox Balance, and Apoptotic Level of Porcine Oocytes

**Figure 3:**
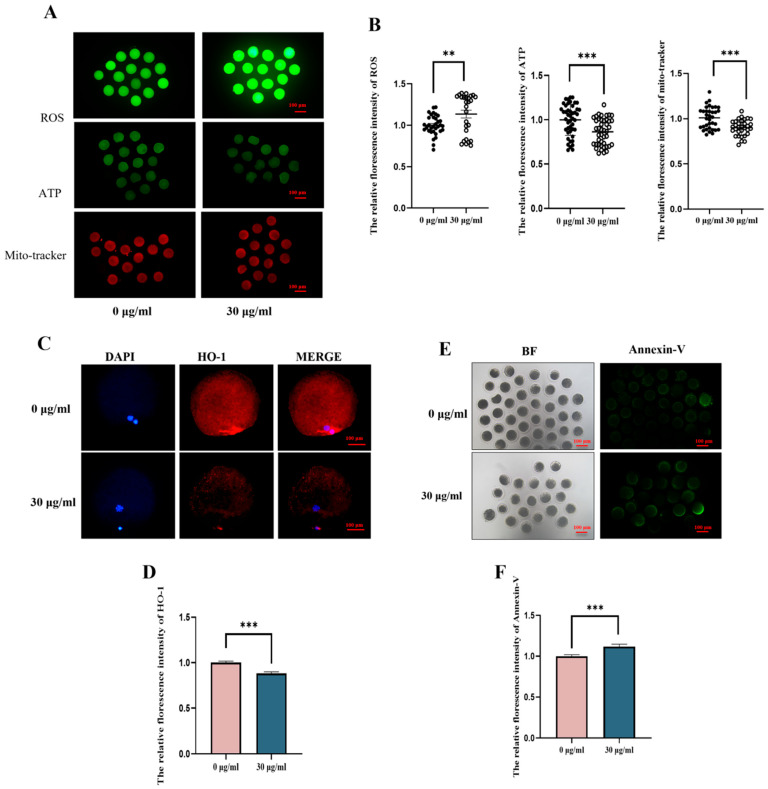
Effects of PS-MPs exposure on mitochondrial function, REDOX balance, and apoptotic levels in porcine oocytes. (**A**) Typical configuration of reactive oxygen species (ROS), ATP, and mitochondrial distribution (Mito-Tracker) in oocytes from the control and 30 µg/mL PS-MPs groups. Scale = 100 µm. (**B**) Relative fluorescence intensity analysis of ROS, ATP, and mitochondrial distribution (Mito-Tracker) levels after exposure to control and 30 µg/mL PS-MPs. **, *p* < 0.01, ***, *p* < 0.001. (**C**,**D**) Representative images and relative fluorescence intensity analysis of HO-1 levels in oocytes from control and 30 µg/mL PS-MPs groups. Scale = 100 µm, *** *p* < 0.01. (**E**,**F**) Representative images and relative fluorescence intensity analysis of Annexin-V levels in oocytes from the control and 30 µg/mL PS-MPs groups. Scale = 100 µm, *** *p* < 0.001. Each oocyte’s average fluorescence intensity was statistically analyzed. Error bars indicated the mean ± SEM. *p*-values were calculated using the *t*-test. The experiment of each group was repeated three times.

Normal mitochondrial function is essential for oocyte maturation. To assess mitochondrial content, Mito-Tracker staining was used. As shown in Figure 3, the mitochondrial fluorescence signal in porcine oocytes from the PS-MPs exposure group was significantly reduced compared to the control group. Fluorescence intensity analysis confirmed this decrease (control: 1.00 ± 0.12, *n* = 16 oocytes vs. PS-MPs: 0.90 ± 0.09, *p* < 0.001, extremely significant, *n* = 15 oocytes, *n* = 3) (Figure 3A,B). To evaluate mitochondrial function further, ATP content was measured. The results showed that oocytes exposed to PS-MPs had significantly lower ATP content compared to the control group, as confirmed by fluorescence intensity analysis (control: 1.00, *n* = 18 oocytes vs. PS-MPs: 0.89 ± 0.03, *n* = 13 oocytes, *p* < 0.001, extremely significant, *n* = 3) (Figure 3A,B). These results indicate that PS-MPs impair mitochondrial function in oocytes, leading to mitochondrial dysfunction.

Mitochondrial function is closely linked to oxidative stress. As depicted in Figure 3A,B, ROS fluorescence signals in the PS-MPs exposure group were significantly higher than those in the control group. Fluorescence intensity analysis confirmed this increase (control: 1.00 ± 0.12, *n* = 14 oocytes vs. PS-MPs: 1.14 ± 0.24, *n* = 15 oocytes, *p* < 0.01, very significant, *n* = 3). Next, the expression level of the antioxidant protein heme oxygenase-1 (HO-1) was assessed through immunofluorescence staining (Figure 3C,D). PS-MPs exposure significantly reduced HO-1 expression in porcine oocytes compared to the control group (control: 1.00 ± 0.11, *n* = 24 oocytes vs. PS-MPs: 0.80 ± 0.09, *n* = 26 oocytes, *p* < 0.001, extremely significant, *n* = 3).

Finally, to verify that oxidative stress and mitochondrial dysfunction induced by PS-MPs exposure contribute to increased apoptosis, Annexin-V staining was performed. As shown in the figure, the Annexin-V fluorescence signal in oocytes exposed to PS-MPs was significantly higher than in the control group. Fluorescence intensity analysis confirmed this increase (control: 1.00 ± 0.17, *n* = 39 oocytes vs. PS-MPs: 1.12 ± 0.23, *n* = 21 oocytes, *p* < 0.001, extremely significant, *n* = 3) (Figure 3E,F).

### 3.3. MT Can Alleviate the Damage to the Meiosis Process of Porcine Oocytes Induced by PS-MPs

**Figure 4:**
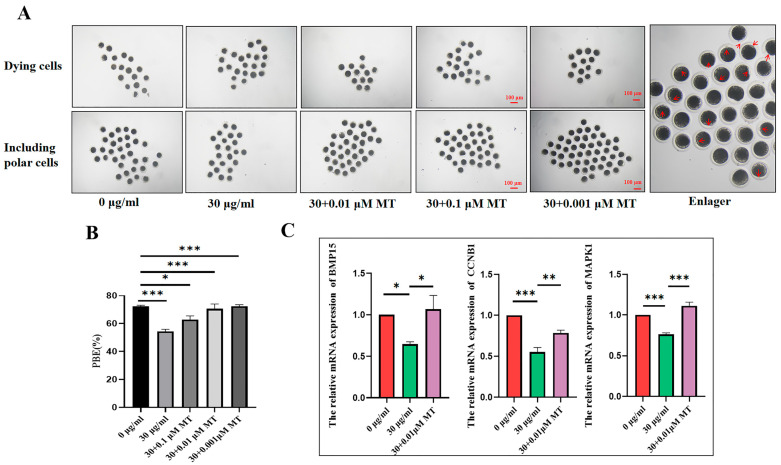
MT alleviates damage to the meiotic process of porcine oocytes induced by PS-MPs. (**A**) Representative morphology of oocyte maturation after treatment with control, 30 µg/mL PS-MPs, and different concentrations of MT. The arrow indicates the polar body extrusion (PBI). Scale = 100 µm. The white arrow in the enlarged image highlights the prominent polar body, used as the standard for polar body selection. The magnification box indicates the selected area for zooming. (**B**) Polar body extrusion rates of the control group, the 30 µg/mL PS-MPs group, and the MT-treated groups at different concentrations. * *p* < 0.05, *** *p* < 0.001. (**C**) Expression of oocyte development-related genes in the control, 30 µg/mL PS-MPs, and MT (0.001 μM) treatment groups. *, *p* < 0.05, **, *p* < 0.01, ***, *p* < 0.001. Each oocyte’s average fluorescence intensity was statistically analyzed. Error bars indicated the mean ± SEM. *p*-values were calculated using the ANOVA test. The experiment of each group was repeated three times.

To evaluate whether MT could alleviate meiotic failure induced by PS-MPs, oocytes were co-cultured with 0.1, 0.01, and 0.001 μM MT and 30 µg/mL PS-MPs, respectively. As shown in the results (Figure 4A,B), all three concentrations of MT significantly increased the proportion of PBI in PS-MP-exposed oocytes to control levels (control: 72.33 ± 1.53%, *n* = 53 oocytes PS-MPs: 54.20 ± 2.82%, *n* = 45 oocytes, MT 0.1 μM: 62.77 ± 4.78%, *n* = 43 oocytes, 0.01 μM: 70.65 ± 6.00%, *n* = 52 oocytes, 0.001 μM: 72.33 ± 1.53%, *n* = 62 oocytes, *n* = 3) (Figure 4B). Based on these findings, 0.001 μM MT was selected for subsequent experiments.

The expression levels of genes associated with oocyte development were further analyzed. Compared to the control group, gene expression related to oocyte development was significantly reduced after exposure to 30 µg/mL PS-MPs. In the MT-treated group, gene expression related to oocyte development significantly increased compared to the PS-MPs group (MAPK1: control: 1.00 ± 0.00, *n* = 76 oocytes vs. PS-MPs: 0.76 ± 0.03, *n* = 76 oocytes, vs. MT: 1.11 ± 0.07, *n* = 76 oocytes, *n* = 3; BMP15: control: 1.00 ± 0.00, *n* = 76 oocytes vs. PS-MPs: 0.78 ± 0.05, *n* = 76 oocytes, vs. MT: 1.12 ± 0.16, *n* = 76 oocytes, *n* = 3; CCNB1: control: 1.00 ± 0.00, *n* = 76 oocytes vs. PS-MPs: 0.55 ± 0.10, *n* = 76 oocytes, vs. MT: 0.78 ± 0.06, *n* = 76 oocytes, *n* = 3) (Figure 4C).

### 3.4. MT Can Alleviate Oxidative Stress and Mitochondrial Damage in Porcine Oocytes Induced by PS-MPs

**Figure 5:**
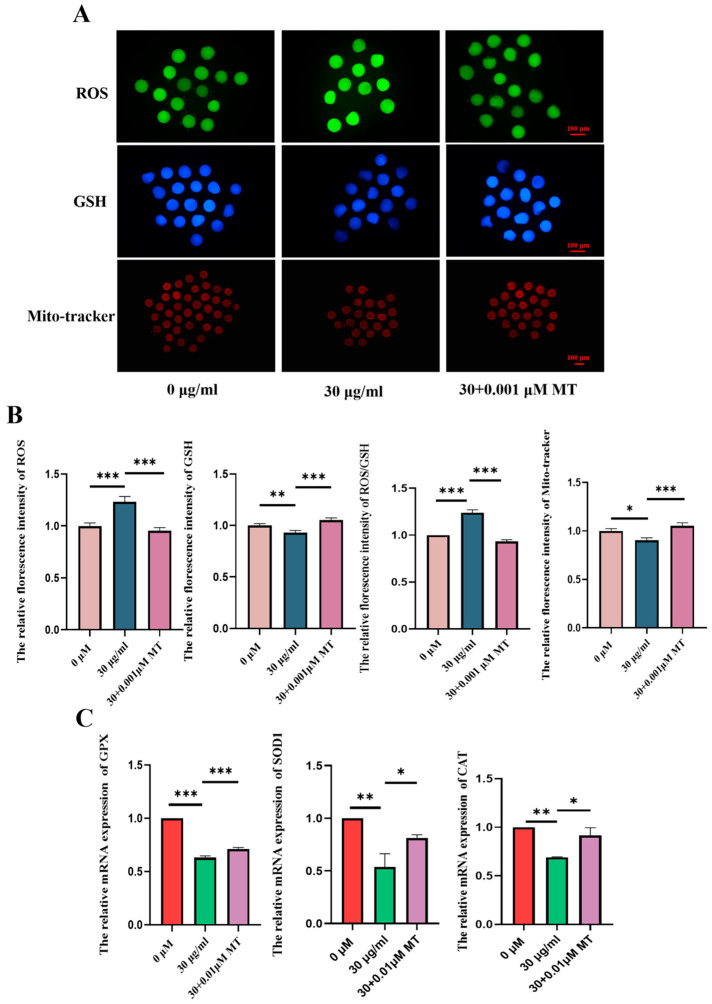
MT alleviates oxidative stress and mitochondrial damage in porcine oocytes induced by PS-MPs. (**A**) Representative images of ROS, GSH, and mitochondrial distribution (Mito-Tracker) levels in oocytes from the control, 30 µg/mL PS-MPs, and MT-treated (0.001 μM) groups. Scale = 100 µm. (**B**) Relative fluorescence intensity analysis of ROS, GSH, ROS/GSH ratio, and mitochondrial distribution (Mito-Tracker) in oocytes treated with control, 30 µg/mL PS-MPs, and 0.001 μM MT. *, *p* < 0.05, **, *p* < 0.01, ***, *p* < 0.001. (**C**) Expression of REDOX-related genes in oocytes from the control, 30 µg/mL PS-MPs, and 0.001 μM MT groups. * *p* < 0.05, ** *p* < 0.01, *** *p* < 0.001. Each oocyte’s average fluorescence intensity was statistically analyzed. Error bars indicated the mean ± SEM. *p*-values were calculated using the ANOVA test. The experiment of each group was repeated three times.

To further confirm whether MT can alleviate the damage induced by PS-MPs, mitochondrial content was first assessed. As shown in Figure 5, the mitochondrial fluorescence signal in porcine oocytes from the MT-treated group was significantly higher compared to the 30 µg/mL PS-MPs group. Fluorescence intensity analysis confirmed this observation (control: 1.00 ± 0.20, *n* = 39 oocytes vs. PS-MPs: 0.90 ± 0.18, *n* = 22 oocytes vs. MT: 1.05 ± 0.21, *n* = 23 oocytes, *n* = 3) (Figure 5A,B).

Next, to evaluate whether MT can reduce oxidative stress in oocytes caused by PS-MPs exposure, the levels of ROS and GSH were measured. As shown in Figure 5, the ROS fluorescence signal in porcine oocytes from the MT-treated group was significantly lower than in the PS-MPs group, while the GSH fluorescence signal was significantly higher. Fluorescence intensity analysis further confirmed these findings (ROS: control: 1.00 ± 0.22, *n* = 15 oocytes vs. PS-MPs: 1.23 ± 0.35, *n* = 11 oocytes, *p* < 0.001, extremely significant vs. MT: 0.95 ± 0.19, *n* = 16 oocytes, *n* = 3; GSH: control: 1.00 ± 0.13, *n* = 18 oocytes vs. PS-MPs: 0.92 ± 0.14, *n* = 15 oocytes vs. MT: 1.05 ± 0.14, *n* = 15 oocytes, *n* = 3) (Figure 5A,B). Analyzing the REDOX balance (ROS/GSH ratio), the ratio in porcine oocytes from the MT-treated group was significantly lower than in the PS-MPs group, indicating a restored REDOX balance (ROS/GSH: control: 1.00 ± 0.00 vs. PS-MPs: 1.24 ± 0.05 vs. MT: 0.93 ± 0.03).

The expression of antioxidant genes, including SOD1, catalase (CAT), and GPX, was measured using real-time fluorescence quantitative PCR. The results showed a significant decrease in the antioxidant genes in oocytes exposed to 30 µg/mL PS-MPs, whereas the MT-treated group showed a significant increase in gene expression (SOD1: control: 1.00 ± 0.00, *n* = 45 oocytes vs. PS-MPs: 0.54 ± 0.22, *n* = 45 oocytes vs. MT: 0.81 ± 0.05, *n* = 45 oocytes *n* = 3; CAT: control: 1.00 ± 0.00, *n* = 45 oocytes vs. PS-MPs: 0.69 ± 0.01, *n* = 45 oocytes vs. MT: 0.92 ± 0.14, *n* = 45 oocytes *n* = 3; GPX: control: 1.00 ± 0.00, *n* = 45 oocytes vs. PS-MPs: 0.63 ± 0.03, *n* = 45 oocytes vs. MT: 0.71 ± 0.03, *n* = 45 oocytes *n* = 3) (Figure 5C).

### 3.5. MT Can Alleviate the Apoptosis and Autophagy Disorders of Porcine Oocytes Induced by PS-MPs

**Figure 6:**
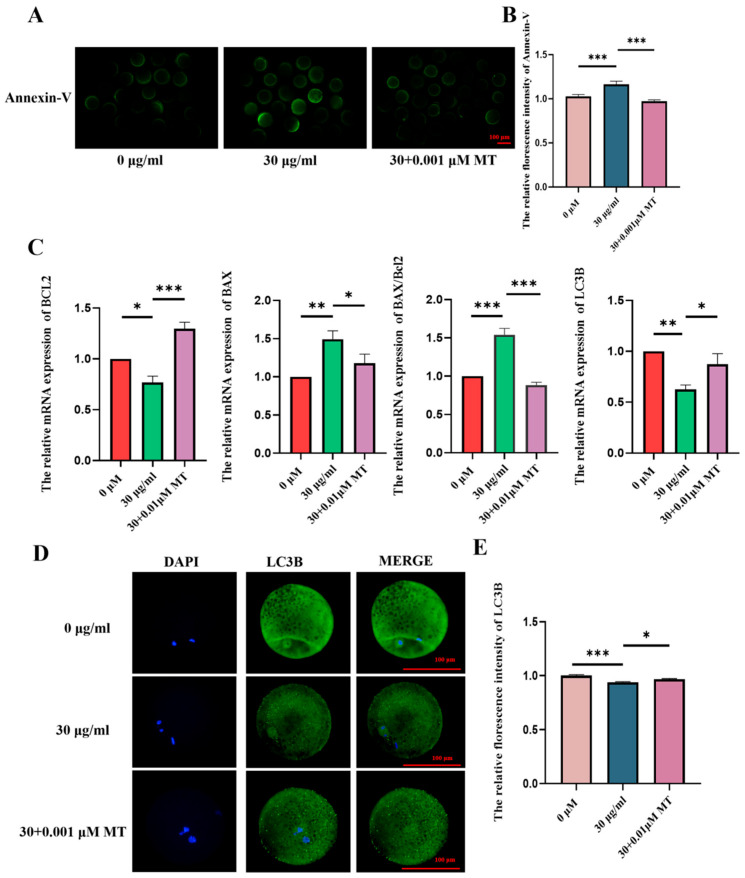
MT alleviates apoptosis and autophagy disorders in porcine oocytes induced by PS-MPs. (**A**) Representative images of Annexin-V levels in oocytes from control, 30 µg/mL PS-MPs, and MT (0.001 μM) treatment groups. Scale = 100 µm. (**B**) Relative fluorescence intensity analysis of Annexin-V levels after treatment with control, 30 µg/mL PS-MPs, and 0.001 μM MT. ***, *p* < 0.001. (**C**) Expression of apoptosis-related genes in oocytes treated with control, 30 µg/mL PS-MPs, and 0.001 μM MT. *, *p* < 0.05, **, *p* < 0.01, ***, *p* < 0.001. (**D**) Representative images of LC3B levels in oocytes from control, 30 µg/mL PS-MPs, and MT (0.001 μM) groups. Scale = 100 µm. (**E**) Relative fluorescence intensity analysis of LC3B levels after treatment with control, 30 µg/mL PS-MPs, and 0.001 μM MT. *, *p* < 0.05, ***, *p* < 0.001. Each oocyte’s average fluorescence intensity was statistically analyzed. Error bars indicated the mean ± SEM. *p*-values were calculated using the ANOVA test. The experiment of each group was repeated three times.

To verify whether MT can alleviate the effects of autophagy and apoptosis induced by PS-MPs exposure, the levels of autophagy-related protein LC3B and early apoptosis-related protein Annexin-V were first assessed. The results showed that, compared to the 30 µg/mL PS-MPs group, the level of LC3B, an autophagy protein, was significantly lower in the MT-treated group. Conversely, the level of Annexin-V, an apoptotic marker, was significantly higher in the MT-treated group compared to the PS-MPs group (Figure 6A,B,D,E). Fluorescence intensity analysis confirmed these observations (LC3B: control: 1.00 ± 0.08, *n* = 74 oocytes, PS-MPs: 0.93 ± 0.05, *n* = 73 oocytes MT: 0.96 ± 0.07, *n* = 72 oocytes, *n* = 3; Annexin-V: control: 1.00 ± 0.17, *n* = 24 oocytes PS-MPs: 1.16 ± 0.30, *n* = 26 oocytes, MT: 0.97 ± 0.13, *n* = 24 oocytes, *n* = 3).

At the genetic level, apoptosis-related genes BAX and BCL2 were significantly modulated in the MT-treated group compared to the PS-MPs group. Additionally, the expression of the autophagy-related gene LC3B was consistent with the fluorescence intensity results. Specifically, BAX and BCL2 expressions were increased in the MT group, while the BAX/BCL2 ratio was significantly reduced, and LC3B expression was significantly improved (BAX: control: 1.00 ± 0.00, *n* = 84 oocytes PS-MPs: 1.35 ± 0.40, *n* = 84 oocytes MT: 1.18 ± 0.27, *n* = 84 oocytes, *n* = 5; BCL2: control: 1.00 ± 0.00, *n* = 84 oocytes PS-MPs: 0.77 ± 0.11, *n* = 84 oocytes, MT: 1.30 ± 0.24, *n* = 84 oocytes, *n* = 3; BAX/BCL2: control: 1.00 ± 0.00, PS-MPs: 1.54 ± 0.15, MT: 0.88 ± 0.06, *n* = 3; LC3B: control: 1.00 ± 0.00, *n* = 84 oocytes, PS-MPs: 0.63 ± 0.08, *n* = 84 oocytes, MT: 0.87 ± 0.18, *n* = 84 oocytes, *n* = 3) (Figure 6C).

### 3.6. PS-MPs Cross Through the NF-KB/Nrf2/JNK MAPK Signaling Pathway to Induce Oxidative Stress, Mitochondrial Damage, and Inflammatory Responses

**Figure 7:**
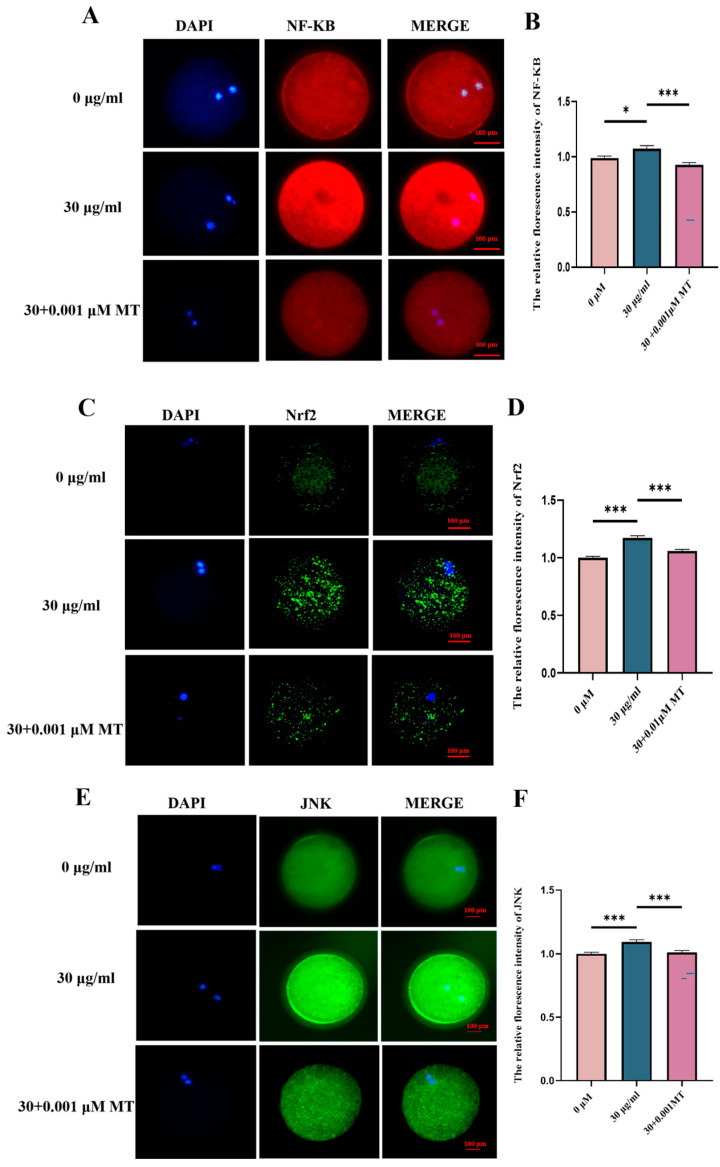
PS-MPs induce oxidative stress, mitochondrial damage, and inflammatory responses via the NF-κB/Nrf2/JNK MAPK signaling pathway. (**A**) Representative images of nuclear factor-protein (NF-κB) levels in oocytes from the control, 30 µg/mL PS-MPs, and MT (0.001 μM) groups. Scale = 100 µm. (**B**) Relative fluorescence intensity analysis of NF-κB levels after treatment with control, 30 µg/mL PS-MPs, and 0.001 μM MT. *, *p* < 0.05, ***, *p* < 0.001. (**C**) Representative images of nuclear factor—erythrocyte factor 2-associated factor 2 (Nrf2) protein levels in oocytes from the control, 30 µg/mL PS-MPs, and MT (0.001 μM) groups. Scale = 100 µm. (**D**) Relative fluorescence intensity analysis of Nrf2 protein levels after treatment with control, 30 µg/mL PS-MPs, and 0.001 μM MT. ***, *p* < 0.001. (**E**) Representative images of C-Jun n-terminal kinase (JNK) protein levels in oocytes from the control, 30 µg/mL PS-MPs, and MT (0.001 μM) groups. Scale = 100 µm. (**F**) Relative fluorescence intensity analysis of JNK protein levels after treatment with control, 30 µg/mL PS-MPs, and 0.001 μM MT. ***, *p* < 0.001. Each oocyte’s average fluorescence intensity was statistically analyzed. Error bars indicated the mean ± SEM. *p*-values were calculated using the ANOVA test. The experiment of each group was repeated three times.

Based on the above findings, ROS was identified as a key signaling factor in the maturation of porcine oocytes induced by PS-MPs. To verify that PS-MPs induce oxidative stress, mitochondrial damage, and inflammatory responses through the NF-κB/Nrf2/JNK MAPK signaling pathway, the protein level of NF-κB was first assessed. Compared to the control group, the NF-κB protein level was significantly higher in the 30 µg/mL PS-MPs group. Furthermore, the NF-κB protein level was significantly reduced in the MT-treated group compared to the PS-MPs group. Fluorescence intensity analysis confirmed this finding (NF-κB: control: 1.00 ± 0.11, *n* = 46 oocytes vs. PS-MPs: 1.07 ± 0.18, *n* = 36 oocytes vs. MT: 0.93 ± 0.14, *n* = 54 oocytes, *n* = 3) (Figure 7A).

Next, downstream markers Nrf2 and JNK, both associated with NF-κB signaling, were measured. Immunofluorescence results revealed that, compared to the control group, the protein levels of Nrf2 and JNK were significantly increased in the 30 µg/mL PS-MPs group, indicating enhanced oxidasstive stress and reproductive toxicity. In contrast, the protein levels of Nrf2 and JNK were significantly reduced in the MT-treated group compared to the PS-MPs group (Nrf2: control: 1.00 ± 0.09, *n* = 67 oocytes vs. PS-MPs: 1.17 ± 0.15, *n* = 51 oocytes vs. MT: 1.06 ± 0.09, *n* = 84 oocytes, *n* = 3; JNK: control: 1.00 ± 0.09, *n* = 57 oocytes vs. PS-MPs: 1.09 ± 0.14, *n* = 47 oocytes vs. MT: 1.01 ± 0.11, *n* = 55 oocytes, *n* = 3) (Figure 7D,F).

### 3.7. MT Can Alleviate the Subsequent Developmental Damage of PS-MPs on the Maturation of Porcine Oocytes—Early Embryos

**Figure 8:**
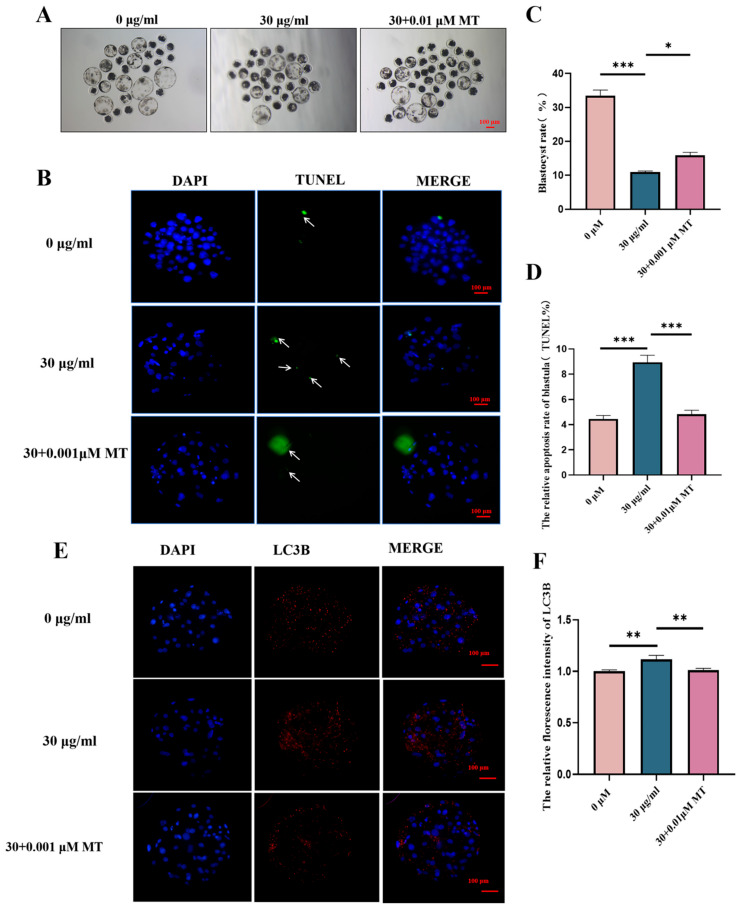
MT alleviates the subsequent developmental damage of PS-MPs to porcine oocytes during maturation and early embryonic development. (**A**) Representative images of early embryonic development in oocytes from the control, 30 µg/mL PS-MPs, and MT-treated (0.001 μM) groups. Scale = 100 µm. (**B**) Relative fluorescence intensity analysis of TUNEL levels in early embryos after treatment with control, 30 µg/mL PS-MPs, and 0.001 μM MT. *, *p* < 0.05, **, *p* < 0.01, ***, *p* < 0.001. (**C**) Statistical analysis of blastocyst formation after treatment with control, 30 µg/mL PS-MPs, and 0.001 μM MT. *, *p* < 0.05, ***, *p* < 0.001. (**D**) TUNEL statistical analysis of blastocyst apoptosis after treatment with control, 30 µg/mL PS-MPs, and 0.001 μM MT. ***, *p* < 0.001. (**E**) Representative images of LC3B levels in oocytes after control treatment. (**F**) Relative fluorescence intensity analysis of LC3B levels in oocytes after treatment with control, 30 µg/mL PS-MPs, and 0.001 μM MT. **, *p* < 0.01. Each oocyte’s average fluorescence intensity was statistically analyzed. Error bars indicated the mean ± SEM. *p*-values were calculated using the ANOVA test. The experiment of each group was repeated three times.

To verify whether MT can alleviate the developmental impairment of porcine oocyte maturation caused by PS-MPs, the early embryo rate was first assessed. Parthenogenetic activation of mature oocytes was performed for each group, and the blastocyst rate was observed. The results showed that the embryo rate of porcine oocytes exposed to PS-MPs was significantly lower than that of the control group. MT treatment improved the early embryo development in PS-MP-exposed oocytes (control: 33.33 ± 2.89%, *n* = 30 oocytes PS-MPs: 10.67 ± 0.57%, *n* = 33 oocytes, MT: 16.00 ± 1.7%, *n* = 38 oocytes, *n* = 3) (Figure 8A).

Next, cell number and apoptosis rate were further assessed using nuclear and TUNEL dye staining. The results showed that the number of cells in porcine oocytes exposed to PS-MPs was significantly lower compared to the control group. MT treatment increased the number of cells in PS-MP-exposed oocytes (control: 49.88 ± 7.62, *n* = 82 Blastocysts vs. PS-MPs: 30.36 ± 6.64, *n* = 42 Blastocysts, vs. MT: 37.04 ± 10.46, *n* = 47 Blastocysts, *n* = 3) (Figure 8C,D). The apoptosis rate of porcine oocytes exposed to PS-MPs was significantly higher than that of the control group, whereas MT treatment reduced the apoptotic rate in PS-MP-exposed oocytes (control: 4.45 ± 1.91%, *n* = 82 Blastocysts, vs. PS-MPs: 8.93 ± 3.21%, *n* = 42 Blastocysts, vs. MT: 4.83 ± 1.67%, *n* = 47 Blastocysts, *n* = 3).

Finally, the autophagy-related protein LC3B in blastocysts from each group was detected. The results showed that the autophagy protein level in porcine oocytes exposed to PS-MPs was significantly higher than in the control group, while MT treatment reduced the autophagy level in PS-MP-exposed oocytes. Fluorescence intensity analysis confirmed this finding (control: 1.00 ± 0.10, *n* = 50 Blastocysts, vs. PS-MPs: 1.12 ± 0.19, *n* = 24 Blastocysts, vs. MT: 1.01 ± 0.11, *n* = 33 Blastocysts, *n* = 3) (Figure 8E,F).

## 4. Discussion

The reproductive toxicity of PS-MPs in mammals remains poorly understood. However, increasing evidence suggests that exposure to PS-MPs adversely affects the reproductive system, leading to disrupted oocyte maturation, oxidative stress, mitochondrial dysfunction, and elevated apoptosis [24,25,26]. Additionally, growing data support that MT can neutralize free radicals, including ROS, a critical factor in reproductive health. MT also plays a significant role in oocyte maturation, fertilization, and embryonic development, including IVM, embryonic development, and survival rates [27,28].

Based on these findings, this study hypothesizes that PS-MPs induce reproductive toxicity and that MT can mitigate PS-MP-induced oocyte damage. To test this, PS-MPs, both alone and combined with MT, were added to in vitro culture systems to assess oocyte maturation rates and investigate the underlying molecular mechanisms.

Exposure to varying concentrations of PS-MPs (0, 25, 50, and 100 μg/mL) in mouse oocytes produced acute toxic effects, including reduced first PBI rates and impaired cumulus cell expansion [28,29]. Similarly, when exposed to 30 μg/mL PS-MPs, porcine oocytes exhibited significantly reduced PBI. However, unlike the reduction in cumulus dilation observed with increasing PS-MPs concentrations in other studies, no difference in cumulus dilation was observed between the PS-MPs groups and the unprocessed group in this study. To provide a more accurate assessment of cumulus dilation, this study categorized dilation into three grades, a statistical approach differing from the method used to assess inhibited cumulus dilation. For subsequent experiments, a concentration of 30 μg/mL PS-MPs was chosen.

In this study, 30 μg/mL PS-MPs induced oxidative stress, a finding consistent with other research indicating that PS-MPs alter sex hormone levels and induce oxidative stress, which may negatively impact fertility and reproduction [30,31]. In the PS-MPs exposure group, ROS levels were significantly elevated, confirming the onset of oxidative stress. Further analysis indicated a substantial reduction in GSH levels, indicating a disruption of the REDOX balance. Oxidative stress was also assessed at the protein level, where immunofluorescence staining showed a significant decrease in HO-1 expression in response to PS-MPs exposure. This result aligns with similar studies demonstrating decreased HO-1 levels in sperm cells following PS-MPs exposure [32]. Additionally, 30 μg/mL PS-MPs induced mitochondrial dysfunction in oocytes. Liu et al. [33] reported that PS-MPs led to reduced mitochondrial membrane potential and ATP content in mouse testicular tissue, with oxidative stress suggested as the underlying cause of mitochondrial damage. Oxidative stress may also accelerate cellular apoptosis. In this study, exposure to 30 μg/mL PS-MPs increased apoptosis in oocytes, which is consistent with findings by An et al. [34], who demonstrated that oxidative stress caused by 25 μg/mL PS-MPs induced apoptosis in ovarian granulosa cells in rats.

MT is not only a key regulator of human reproductive function but also plays an important role in promoting positive pregnancy outcomes [35]. Additionally, MT can mitigate damage caused by MPs. For instance, El Gazzar et al. [36] found that MT alleviated the harmful effects of polyethylene MPs on the intestinal barrier in albino rats. The current study supports the hypothesis that MT can alleviate oocyte meiotic failure and oxidative stress induced by PS-MPs. Similar studies have demonstrated that MT supplementation effectively reduces developmental toxicity and oxidative damage in offspring; however, the detrimental effects of PS-MPs cannot be entirely eliminated [37]. The present study contributes new insights by demonstrating that MT can alleviate mitochondrial dysfunction in germ cells induced by PS-MPs, filling a critical gap in the existing literature.

This study further confirmed that MT can mitigate apoptosis and autophagy disorders during oocyte maturation induced by PS-MPs. Apoptosis, a form of cell death dependent on ROS, was also found to be induced by PS-MPs. Zeng et al. [37] demonstrated that PS-MPs caused oxidative stress in offspring, impaired the immune system, reduced antioxidant capacity, and triggered apoptosis, which aligns with the findings of this study. In contrast, a study on environmental MPs (EMP) showed that PS-MPs increase hepatocyte autophagy, while MT alleviates this autophagy to counteract PS-MP-induced damage [38]. The discrepancy with this study may be attributed to the use of germ cells, which exhibited distinct molecular responses. The dual effects of autophagy warrant further investigation. Nonetheless, the current study indicates that MT can alleviate cellular dysfunction induced by EMP; this is consistent with previous research findings.

This study investigated the oxidative stress induced by PS-MPs in porcine oocytes, highlighting ROS as a key influencing factor. The protein level analysis revealed that mitochondrial dysfunction, apoptosis, and autophagy in porcine oocytes are closely linked to the elevation of ROS. High ROS levels produced during oxidative stress lead to the release of inflammatory mediators. NF-κB activity is also regulated by ROS levels, with ROS having the capacity to both activate and inhibit NF-κB signaling, depending on the environment [39,40,41]. PS-MPs exposure resulted in increased ROS content in porcine oocytes, and protein level analysis showed a significant rise in NF-κB expression. Following MT treatment, NF-κB levels were significantly reduced, suggesting that ROS overload activates NF-κB, an inflammatory factor. Similar research by Shengchen et al. further supports these findings, showing that PS-MPs exposure exacerbates ROS production by upregulating NF-κB expression and promoting adipogenic differentiation [42].

Nrf2 is a critical regulator in reaction to oxidative stress, while NF-κB is a key factor in regulating inflammatory and immune responses. Under physiological conditions, these two transcription factors mutually restrict each other to maintain intracellular redox balance [43]. The results of this study show a significant increase in Nrf2 levels following PS-MPs exposure, while MT treatment significantly reduced Nrf2 expression. The simultaneous elevation of NF-κB and Nrf2 levels confirmed the REDOX imbalance in porcine oocytes exposed to PS-MPs. Furthermore, the level of HO-1 was significantly decreased under PS-MPs exposure, confirming the presence of oxidative stress. However, MT at this concentration did not restore HO-1 levels. Similar studies have reported that PS-MPs exposure in mouse sperm resulted in increased expression of the pro-inflammatory molecule NF-κB and decreased levels of the anti-inflammatory molecule Nrf2/HO-1 [32].

One anti-apoptotic function of NF-κB is its ability to downregulate JNK activation. Further research has shown that NF-κB inhibits JNK activation by suppressing ROS accumulation [44]. Consistent with these findings, the current study observed significantly elevated levels of ROS, NF-κB, and JNK in porcine oocytes exposed to PS-MPs. MT treatment, however, significantly alleviated these effects, reducing reproductive toxicity. Additionally, the JNK/c-Jun cascade induces cell cycle arrest and programmed cell death, including apoptosis and autophagy. Li et al. [45] demonstrated that treatment with ROS scavengers and JNK inhibitors significantly reduced the apoptosis and autophagy induced by the ROS-dependent JNK/c-Jun cascade in metformin-treated cells.

These results suggest that PS-MPs may induce oxidative stress, mitochondrial damage, and inflammatory responses through the NF-κB/Nrf2/JNK MAPK signaling pathway. Moreover, MT inhibits excessive ROS production, preventing PS-MP-induced oxidative stress and mitochondrial damage, alleviating inflammation, and reducing reproductive toxicity.

MPs can enter the human body through direct physical contact or via the food chain, increasing the potential for adverse effects on pregnancy and fetal development. This study examined the subsequent developmental capacity of oocytes through parthenogenetic activation of embryos. The results showed a significant reduction in the development of oocytes exposed to PS-MPs, as evidenced by a marked decrease in the blastocyst rate, autophagy disorders, and increased apoptosis. However, MT significantly alleviated these adverse effects. Similar findings have been reported, indicating that PS-MPs can cause fetal growth restriction in the human placenta and fetus. The mechanisms have been further elucidated, with studies suggesting that PS-MPs activate autophagy, inhibit trophoblast cell migration/invasion, and impair migratory formation, leading to miscarriage. Additional research has shown that PS nanoparticles induce excessive ROS production and apoptosis in neuronal cell lines, while antioxidant supplementation, such as GSH, can prevent these effects [46,47,48]. These studies corroborate the findings of this study. However, further in vivo research is needed.

Notably, the autophagy level in porcine oocytes exposed to PS-MPs was found to decrease during the maturation stage, while it increased during the embryonic stage. MT was able to improve these conditions. A study published in Cell supports this observation, showing that in mouse oocytes, the number of “large LC3 sites” (possibly ELas-ELVA, a non-membrane-bound chamber composed of endolysosomes, autophagosomes, and proteasomes, bound together by a protein matrix formed by RUFY1) increases with age [49]. Exploring protein degradation in oocytes and its regulation offers an exciting avenue for explaining age-related declines in embryonic health [49].

This study sheds light on the potential mechanisms of reproductive toxicity in porcine oocytes exposed to PS-MPs, and it provides new insights into the protective role of MT in mitigating these effects. The findings offer theoretical support and potential solutions for addressing the reproductive toxicity caused by exposure to the environmental pollutant PS-MPs.

## 5. Conclusions

**Figure 9:**
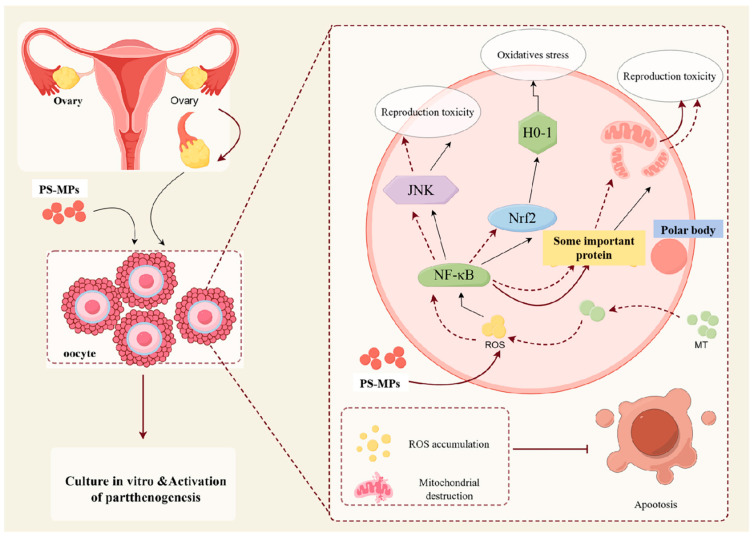
The influence of PS-MPs on oocyte maturation. Dashed arrows indicate that MT alleviates the effects of PS-MPs, while solid arrows show the changes in cell-related indicators in the PS-MPs treatment group. (By Figdraw, https://www.figdraw.com, accessed on 25 March 2025).

In conclusion, this study presents novel insights into early embryonic apoptosis and autophagy during the subsequent development of mammals, focusing on the toxic effects of PS-MPs on oocyte maturation. For the first time, the effect and mechanism of MT in mitigating the toxic impact of PS-MPs in mammals were investigated. Exposure to 30 μg/mL PS-MPs resulted in a significant reduction in the first polar body extrusion rate and the expression levels of genes related to oocyte development. Additionally, PS-MPs exposure induced mitochondrial dysfunction, apoptosis, autophagy, and oxidative stress. The results suggest that PS-MPs may affect these processes via the NF-κB/Nrf2/JNK MAPK signaling pathway, leading to oxidative stress, mitochondrial damage, and inflammation. However, MT treatment alleviated these damages. Moreover, PS-MPs exposure reduced the embryonic developmental potential, but MT treatment successfully rescued the developmental damage to porcine oocyte maturation caused by 30 μg/mL PS-MPs, thereby reducing reproductive toxicity in early embryos (Figure 9).

In terms of research methodology, advanced techniques such as Western blotting were not fully utilized due to challenges in sample collection. As for the research subject, pig oocytes, the use of large animals like pigs entails high costs, time, and complexity, thus necessitating further in vivo studies. The author emphasizes the importance of addressing atmospheric MP pollution through methods like capture technologies or biodegradation processes, urging the protection of environmental health. This highlights the urgent need for more environmentally friendly and sustainable products to safeguard both ecological and human health.

## Figures and Tables

**Table 1:** Primer sequences for RT-qPCR.

Genes	Forward Primers (5′ to 3′)	Reverse Primers (5′ to 3′)
*GAPDH*	GTCGGTTGTGGATCTGACCT	TTGACGAAGTGGTCGTTGAG
*PTX3*	TGTGTGGGTGGTGGCTTTGATG	TGGGGCTGAATCTCTGTGACTCC
*GDF9*	CAGTCAGCTGAAGTGGGACA	TGGATGATGTTCTGCACCAT
*ERK1*	CCTCCAACCTGCTCATCAACAC	ACATATTCCGTCAAGAAGCCAGTG
*MAPK1*	GGCTGTTCCCAAATGCTGACTC	CCTGCTCTACTTCAATCCTCTTGTG
*CAT*	AGCCAGTGACCAGATGAAGCATTG	ATGTCGTGTGTGACCTCAAAGTAGC
*GPX*	CTGGTCGTGCTCGGCTTCC	GCCTGGTCGGACGTACTTGAG
*SOD1*	CTCTCGGGAGACCATTCCATCATTG	TCCACCTCTGCCCAAGTCATCTG
*BAX*	TGCCTCAGGATGCATCTACC	AAGTAGAAAAGCGCGACCAC
*BCL2*	AGGGCATTCAGTGACCTGAC	CGATCCGACTCACCAATACC
*CDK1*	GGGCACTCCCAATAATGAAGT	GTTCTTGATACAACGTGTGGGAA
*BMP15*	CCTCCATCCTTTCCAAGTCA	GTGTAGTACCCGAGGGCAGA
*LC3B*	GTGTAGTACCCGAGGGCAGA	TTTGGTAGGATGCTGCTCTCG

## Data Availability

The data presented in this study are available upon request from the corresponding author.
